# Enzymatic Remediation of Polyethylene Terephthalate (PET)–Based Polymers for Effective Management of Plastic Wastes: An Overview

**DOI:** 10.3389/fbioe.2020.602325

**Published:** 2020-11-19

**Authors:** Ankita Maurya, Amrik Bhattacharya, Sunil Kumar Khare

**Affiliations:** Enzyme and Microbial Biochemistry Laboratory, Department of Chemistry, Indian Institute of Technology Delhi, New Delhi, India

**Keywords:** plastic pollution, polyethylene terephthalate, remediation, PET hydrolases, recycling

## Abstract

Globally, plastic-based pollution is now recognized as one of the serious threats to the environment. Among different plastics, polyethylene terephthalate (PET) occupies a pivotal place, its excess presence as a waste is a major environmental concern. Mechanical, thermal, and chemical-based treatments are generally used to manage PET pollution. However, these methods are usually expensive or generate secondary pollutants. Hence, there is a need for a cost-effective and environment-friendly method for efficient management of PET-based plastic wastes. Considering this, enzymatic treatment or recycling is one of the important methods to curb PET pollution. In this regard, PET hydrolases have been explored for the treatment of PET wastes. These enzymes act on PET and end its breakdown into monomeric units and subsequently results in loss of weight. However, various factors, specifically PET crystallinity, temperature, and pH, are known to affect this enzymatic process. For effective hydrolysis of PET, high temperature is required, which facilitates easy accessibility of substrate (PET) to enzymes. However, to function at this high temperature, there is a requirement of thermostable enzymes. The thermostability could be enhanced using glycosylation, immobilization, and enzyme engineering. Furthermore, the use of surfactants, additives such as Ca^2+^, Mg^2+^, and hydrophobins (cysteine-rich proteins), has also been reported to enhance the enzymatic PET hydrolysis through facilitating easy accessibility of PET polymers. The present review encompasses a brief overview of the use of enzymes toward the management of PET wastes. Various methods affecting the treatment process and different constraints arising thereof are also systematically highlighted in the review.

## Introduction

Indiscriminate usage of plastics and related products along with its poor disposal management leads to the widespread presence of plastic waste in the environment. The need for plastic becomes so much prevalent that it is now described as one of the inseparable commodities (Koshti et al., 2018). Various properties such as light weight, heat resistance, high malleability, transparency, hardness, and tensile strength make plastics as one of the desirable polymers for a variety of applications. This extensive application of plastic resulted in the steady rise of plastic waste in different ecosystems. Plastic is highly recalcitrant and takes about 1,000 years to decompose in nature, and thus, it keeps on accumulating in nature (Webb et al., 2013). This excess accumulation of plastic and associated wastes in the environment possesses various risks to living beings (Ogunola et al., 2018; Saleem et al., 2018).

From the start of the 21st century, the production of plastic has increased tremendously because of high demand, and as a consequence of it, plastic waste generation also tripled in these two decades (Beat Plastic Pollution, 2020). At present, around 0.3 billion of plastic wastes are produced, and 90% of it lands up in the ocean (Schmidt et al., 2017). Since the 1950s, around 8,300 million plastic wastes has been generated, and by 2050, it is expected to reach double-digit billions, if plastic waste is generated at the same pace (Geyer et al., 2017).

Plastics have resistance toward organic solvents, oxidation, and ionizing radiation, making it a priority choice for many industrial applications. From the total plastic production, 33% are used in packaging (Rhodes, 2018). Among various forms of plastics, polyethylene terephthalate (PET)–based plastics are noteworthy as they are widely used in packaging industries because of their durability and thermostability. PET is a semicrystalline, colorless, hygroscopic resin with excellent properties of high wear and tear resistance, high tensile strength, and transparency (Koshti et al., 2018). Owing to these properties, PET is extensively used in packaging industries. Prominently, it is used in plastic bottles of soft drinks, food jars, and plastics films.

Polyethylene terephthalate is synthesized by polycondensation of terephthalic acid (TPA) and ethylene glycol (EG) or transesterification of dimethyl terephthalate and EG forming a polymer of semiaromatic polyesters (Hiraga et al., 2019). TPA and EG monomeric units of PET are linked by ester linkages. It is chemically inert and hydrophobic in character, which creates an almost non-soakable surface (de Castro et al., 2017). Melting temperature (*T*_m_) of PET is noted to be 240–250°C with good hydrolytic stability (Mohsin et al., 2017). PET varies in crystallinity (CrI); for instance, those possessing up to 7% CrI are called low crystalline PET (lcPET) and with 30–35% CrI is high-crystalline PET (hcPET) (Furukawa et al., 2019). The extent of CrI depicts the mobility of ester linkages in PET (Zekriardehani et al., 2017). High CrI indicates more rigidity in the linkages. The glass transition temperature (*T*_g_) of PET is around 70–80°C (Ronkvist et al., 2009). *T*_g_ is the temperature at which mobility of the polymer is increased, allowing more accessibility to ester links between monomeric units.

The high demand for PET-based plastics, especially in packaging industries, leads to the total production of 18.8 million tons in 2015 out of total of 269 million tons of total plastic production (Taniguchi et al., 2019). Out of total PET production, only 28.4% is recycled to fiber, sheets, films, and bottles, and the rest is discarded in the environment (Taniguchi et al., 2019). This discarded PET then goes into the open environment and forms a threat to the various life forms.

Like other plastics, PET is usually non-biodegradable and difficult to decompose, especially those with high crystallinity. Hence, most of the PET-based plastic wastes are either incinerated or dumped in landfill sites (Geyer et al., 2017). A very low portion of it is recycled. As mentioned above, accumulation of plastic wastes affects the normal functioning of an ecosystem through various detrimental effects on living forms. Consumption of plastic materials by stray animals and tiny plastic materials floating on water bodies by aquatic animals lead to various alterations in their physiological activities (Bhattacharya and Khare, 2020). Sometimes, this consumption also leads to blockage of the digestive system and clogging of respiratory passage and ultimately results in the mortality of particular animal species (Koshti et al., 2018). In addition to that, the toxic constituents released during the partial decomposition of plastic wastes also add on to soil pollution and affect various life forms. As PET-based plastics are hydrophobic in nature and thus act as adsorption sites for various pollutants such as persistent organic pollutants and heavy metals found in aquatic and terrestrial systems (Bhattacharya and Khare, 2020). These adhered toxins are also transferred through the food chain and possess a risk for high-trophic-level consumers, as these may get biomagnified upon transfer through food chains (Koshti et al., 2018).

At present, majorly employed plastic/PET disposal methods in developing countries are landfilling and incineration. Landfilling cannot be carried out for long because of scarcity of space and increasing cost; similarly, incineration results in an emission of toxic fumes containing various toxicants and fly ash, which requires further disposal (Saleem et al., 2018). However, recycling is considered one of the best ways to manage plastic/PET wastes. Recycling uses less energy and fewer resources and also leads to the lowering of carbon footprint compared to the production of petrochemical-based virgin PET products (Quartinello et al., 2017). Post consumption, PET wastes in many countries (most of the European countries and Japan) are recycled to form new products through the recovery of PET monomers (TPA and EG). Various recycling options *viz.* thermal (used as fuels), material/mechanical (melted and reused once), and chemical/catalytic (degraded to monomers and used for re-synthesis) are usually practiced for management of PET wastes (Kawai et al., 2019).

Material or mechanical recycling is one of the widely used methods for recycling of PET wastes. This process involves sorting and separation of wastes followed by washing for the removal of dirt and contaminants from wastes (Park and Kim, 2014). Thereafter grinding and crushing are accomplished to reduce the particle size; finally, reextrusion and reprocessing are done for the production of new products (Park and Kim, 2014). However, heterogeneity of waste along with the presence of contaminants mainly hinders mechanical processing. Additionally, products formed from mechanically recycled PET wastes are of poor quality because of mechanical stress and photo-oxidation caused by the heat of fusion (Park and Kim, 2014; Kawai et al., 2019). Hence, this method of recycling is generally practiced for the production of low-grade plastics. Similarly, thermal processing/incineration of PET waste is considered to be undesirable as it results in air pollution through the generation of toxic fumes.

Chemical-based recycling involves the degradation of PET into its monomeric, oligomeric, and other chemical forms using various processes involving different harsh chemicals (Park and Kim, 2014). Glycol-based glycolysis, hydrolysis using strong acids and alkali, and aminolysis using primary amines are some of the chemical-based recycling methods (Joo et al., 2018). These methods are not environment-friendly and cost-effective and thus are normally not suggested for the recycling of PET wastes.

**FIGURE 1:**
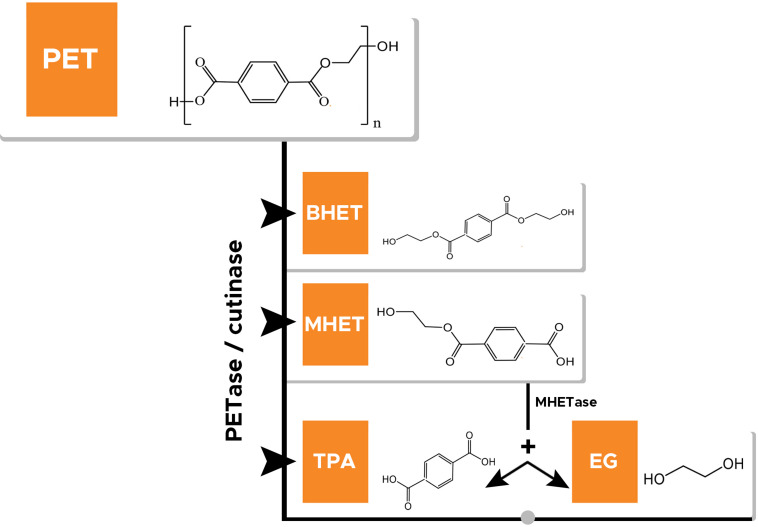
Enzyme catalyzing depolymerization of polyethylene terephthalate (PET) into bis(hydroxyethyl)terephthalate (BHET), mono(hydroxyethyl)terephthalate (MHET), terephthalic acid (TPA), and ethylene glycol (EG). MHETase catalyzes hydrolysis of MHET to TPA and EG.

In the midst of the aforementioned recycling methods, biocatalytic-based recycling of PET is identified as one of the efficient and eco-friendly strategies for the management of PET wastes. Biocatalytic recycling not only sustainably manages the PET wastes, but also the products formed through this process possess the same properties as the virgin PET. PET hydrolases are identified to play a pivotal role in catalytic-based recycling of PET wastes. However, considering the total PET production, still the total recycling or recovery rate is very low, and most of these wastes enter into landfills and open aquatic environment. Catalytic-based recycling of PET waste is successfully implemented in many countries (France, Japan) and identified as one of the contributing factors in the concept of a circular PET economy (Tournier et al., 2020). Nevertheless, efficient PET hydrolases are very limited in number, and still, only four PET hydrolases (cutinases) have been identified that can significantly degrade PET to its monomers (Kawai et al., 2020). However, other specialized enzymes, *viz*. lipase, esterase, and PETase, have been used by several researchers for the hydrolysis of PET into monomeric forms *viz.* TPA, EG, mono-2-hydroxyethyl terephthalate (MHET), and *bis*(2-hydroxyethyl) terephthalate (BHET) (Wang et al., 2008; Ribitsch et al., 2011; Ma et al., 2012; Bollinger et al., 2020). Figure 1 represents a schematic diagram showing various recycling methods for PET with associated processes and their outcomes.

In this review, a brief overview of enzymatic recycling of PET wastes and different methods used to enhance the recycling performance are discussed. Various factors affecting the hydrolysis rate and constraints arising during the hydrolysis process are also highlighted in the review.

## Biological Approach for Management of PET Wastes

**FIGURE 2:**
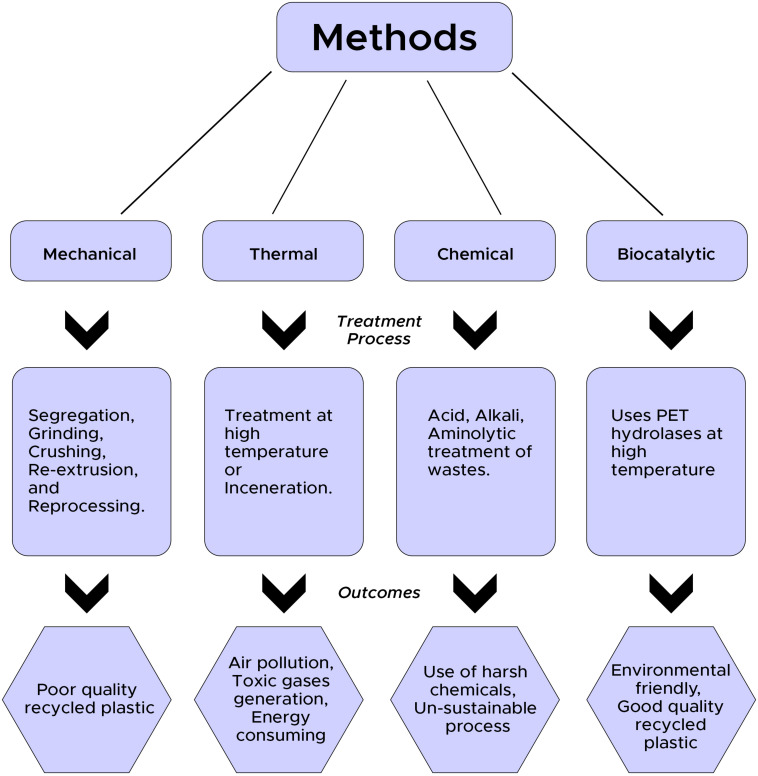
Methods used to recycle PET wastes.

The high recalcitrant nature of plastic waste including PET waste is a major bottleneck; however, biological recycling involving enzymatic-hydrolysis of plastic may be used to tackle the menace of plastic/PET pollution in an eco-friendly and efficient way. As mentioned above, PET is linked by ester bonds, which can be hydrolyzed by various hydrolytic enzymes into its monomers TPA, EG, MHET, and BHET (Ronkvist et al., 2009; Ribitsch et al., 2011). Figure 2 shows the enzymatic hydrolysis of PET. But ester linkages of PET usually have low accessibility and thus become difficult for depolymerization (Austin et al., 2018). However, enzymes possessing esterase/hydrolytic activity have been used widely for the hydrolysis of PET. Table 1 shows the list of various enzymes (PET hydrolases) reported to hydrolyze PET.

**TABLE 1:** Microbial enzymes known to hydrolyze PET.

Enzymes	Microorganisms	References
1. PETase	*Ideonella sakaiensis*	Yoshida et al., 2016
2. Cutinase	*Thermobifida fusca*	Müller et al., 2005
	*Humicola insolens*	Ronkvist et al., 2009
	*Thermobifida cellulosilytica*	Herrero Acero et al., 2011
	*Thermobifida alba*	Ribitsch et al., 2012
	*Fusarium solani pisi*	Sulaiman et al., 2012
	*Saccharomonospora viridis*	Kawai et al., 2014
	*Fusarium oxysporum*	Dimarogona et al., 2015
	*Aspergillus fumigatus*	Ping et al., 2017
3. Lipase	*Triticum aestivum*	Nechwatal et al., 2006
	*Thermomyces lanuginosus*	Eberl et al., 2009
4. Carboxylesterases	*Thermobifida fusca*	Billig et al., 2010
5. Polyester hydrolase	*Thermomonospora curvata*	Wei et al., 2014
	*Pseudomonas aestusnigri*	Bollinger et al., 2020

Although various enzymes are reported to degrade PET naturally, the extent of degradation is found to be quite low. Some esterases, for example, PETase (Austin et al., 2018), cutinase (Sulaiman et al., 2012), and lipase (Macedo and Pio, 2005), have been used to hydrolyze BHET to yield MHET. MHET can further enzymatically degrade using MHETase into TPA and EG (Koshti et al., 2018). Factors such as the crystallinity of PET, hydrophobicity, and structure usually limit enzyme function (Koshti et al., 2018). These and other factors are discussed later in the review.

Interest in PET hydrolases gains momentum with the work of Yoshida et al. (2016). Yoshida and coworkers reported a novel bacterium *Ideonella sakaiensis* 201-F6 capable of using low-crystallinity (1.9%) PET film as a major carbon and energy source. *I. sakaiensis*, when grown on the PET, secretes PET-hydrolyzing enzymes (PETase, MHETase), which synergistically hydrolyze PET polymers into monomeric forms. PETase is a key enzyme for PET degradation, and cutinase has shown maximum similarity with PETase (Liu et al., 2018). But PETase has a broader active site compared to cutinase, making high accommodative region for PET (Chen et al., 2018). As cutinases are found in eukaryotes and prokaryotes, it is widely available for biodegradation. In this review, cutinase is covered to a larger extent because of its wide coverage in literature toward PET degradation.

### Enzymes Used for PET Hydrolysis

#### Cutinase

Cutinase (E.C. 3.1.1.74) is majorly produced by either saprophytic microorganism, which utilizes cutin as a carbon source or by phytopathogenic microorganisms for breaking the cutin barrier to enter into the host plants. Cutinase is a serine esterase that has the catalytic triad consisting of Ser–His–Asp residues (Egmond and de Vlieg, 2000). It belongs to the α/β hydrolase superfamily (Egmond and de Vlieg, 2000). The active site of cutinase can accommodate high-molecular-weight compounds such as cutin and other related synthetic compounds. Hydrolysis of synthetic polymers such as PET (Dimarogona et al., 2015), polycaprolactone (Adıgüzel and Tunçer, 2017), polystyrene (PS) (Ho et al., 2018), polyethylene furanoate (Weinberger et al., 2017), and polybutylene succinate (Hu et al., 2016) have also been reported using cutinase. Cutinase-mediated hydrolysis of polylactic acid is also demonstrated by several authors (Masaki et al., 2005; Kitadokoro et al., 2019).

Cutinase is widely reported from fungal and bacterial species. Table 2 enlists some of these cutinases with their respective PET hydrolysis rates. The catalytic efficiency of cutinase has been observed maximum with *p*-nitrophenyl butyrate and *p*-nitrophenyl acetate (Herrero Acero et al., 2011; Yang et al., 2013), and thus, it has more affinity for the substrate with less carbon chain length compared to other substrates.

**TABLE 2:** Effect of temperature on enzymatic PET hydrolysis.

Cutinase/hydrolase from microorganism	Optimum temperature for PET degradation	PET degradation rate	References
*Humicola insolens*	80°C	97% with lcPET with 7% crystallinity	Ronkvist et al., 2009
*Pseudomonas mendocina*	50°C	5% with lcPET with 7% crystallinity	Ronkvist et al., 2009
*Fusarium solani*	40°C	5% with lcPET with 7% crystallinity	Ronkvist et al., 2009
*Thermobifida cellulosilytica*	50°C	24% with semicrystalline PET and 12% with lcPET	[Bibr B32][Bibr B25][Bibr B26]
*Thielavia terrestris*	50°C	–	Yang et al., 2013
*Thermobifida alba* (Tha_cut1)	50°C	–	Ribitsch et al., 2012
*Candida antarctica* lipase B (CalB)	37–50°C	–(hydrolyze BHET to TPA)	[Bibr B14][Bibr B9]
Leaf and Branch compost cutinase	75°C	25% in 24 h (LCC -NG 95% in 48 h (LCC -G)	Shirke et al., 2018
*Thermobifida fusca T. fusca* KW3	55°C–60°C 60°C–65°C	50% with lcPET crystallinity of 9% 15.9% ± 1.8% with amorphous PET	[Bibr B56][Bibr B18][Bibr B74][Bibr B95]
(TfCut2) Tfu_0883/Tfu_0882	40°C or 60°C	–	[Bibr B83][Bibr B13]

Owing to the versatility of hydrolyzing a broad range of ester bonds and to catalyze esterification and transesterification reactions, cutinase is viewed as a promising enzyme for various industrial applications. For instance, it is widely used in the field of oil and dairy products, flavor compounds, and phenolic compounds production (Dutta et al., 2009; Chen et al., 2013). It is also reported in insecticide and pesticide degradation (Dutta et al., 2009; Chen et al., 2013). As an enzyme, it has not only been studied for the degradation of polyesters but has also been used in fiber modification (Alisch et al., 2004). Cutinase possesses valuable properties particularly required for PET degradation, and thus, it has caught the eye of many researchers in recent years. It is a well-studied substitute for harsh chemicals usually practiced during chemical-based hydrolysis/recycling of plastics (Macedo and Pio, 2005; Tournier et al., 2020). Cutinases are also known to synthesize polyesters under non-aqueous media using polycondensation reaction with various diacids and alcohols. In this aspect, cutinase from *Humicola insolens* (HiC) immobilized on Lewatit beads was used by Hunsen et al. (2007) for the synthesis of polyester through the condensation reaction. Similarly, Pellis et al. (2016) used cutinase 1 from *Thermobifida cellulosilytica* for polycondensation of dimethyl adipate with various polyols for the synthesis of high-molecular-weight polyesters.

#### Lipase

Lipase has also been used by several researchers for the hydrolysis of PET. Effective degradation of PET nanoparticles using lipase from *Candida cylindracea* and *Pseudomonas* sp. has been reported by Ma et al. (2012). Similarly, Wang et al. (2008) employ BHET/TPA-induced lipase from *Aspergillus oryzae* for hydrolysis of PET. Moreover, Carniel et al. (2017) and de Castro et al. (2017) used the combination of lipase from *Candida antarctica* (*C. antarctica* lipase lB CALB) and HiC for efficient PET hydrolysis to TPA. Although HiC showed better performance with PET hydrolysis, the enzyme has limited competence to convert MHET (one of the intermediates of PET hydrolysis) into TPA. On the other hand, CALB can easily convert MHET into TPA but has lower efficiency toward initial PET hydrolysis when used singly. However, the combination of both enzymes synergistically improves the overall PET hydrolysis. However, complete studies on the effect of enzyme dosages, temperature, and pH are lacking. Lipase and cutinase have a common feature of surface hydrophobicity (de Castro et al., 2017). Unlike other lipases, lipase B has a superficial catalytic site; hence, in the absence of the hydrophobic interface, it is still accessible to the substrate (Stauch et al., 2015).

#### Esterase

Monomers of PET are linked by ester linkage, and these can be cleaved using esterase found in almost all living organisms (Koshti et al., 2018). Ribitsch et al. (2011) used *Bacillus subtilis* nitrobenzylesterase (BsEstB) and applied it to hydrolyze PET into TPA and MHET [mono(2-hydroxyethyl)] TPA. Kawai et al. (2014) made use of recombinant thermostabilized polyesterase from *Saccharomonospora viridis* AHK190 capable of hydrolyzing PET and the PET-hydrolyzing activity was observed to increase in presence of Ca ions. Recombinant esterase from *Thermobifida halotolerans* (Thh_Est) was reported by Ribitsch et al. (2012) to degrade PET into TA and MHET.

#### PETase

PETase (3.1.1.101) was discovered from the bacterium *I. sakaiensis* 201-F6 by Yoshida et al. (2016). PETase and cutinases share high sequence identity, indicating the existence of critical structural features responsible for substrate binding (Fecker et al., 2018; Kawai et al., 2019). Even small differences between these enzymes are crucial and define their specific activities (Chen et al., 2018). High-resolution crystal structure study of PETase highlights the active site, which seems to be wider than the other cutinases, and thus this could be a factor of the high specificity of the enzyme toward heavy substrate PET (Chen et al., 2018; Kawai et al., 2020).

Overall, PET hydrolases (PET-hydrolyzing enzymes) are generally limited to cutinases; structurally, they are homologous to lipase, but lack a lid covering the active site (Kawai et al., 2019). This shallow open active site with hydrophobic amino acid residues aids in PET binding and hydrolysis (Kawai et al., 2020). The lid is present in the active site of lipase and is known for interfacial activation in lipases. Lipases are not much active in PET hydrolysis, but like esterases and cutinases, they are known for surface modification of PET fibers. Esterase activity is limited to short-chain acyl esters and thus is also not much reported to hydrolyze hydrophobic PET. Comparative X-ray crystallography data of actinomycetes cutinases and PETase (from *I. sakaiensis*) showed the presence of a broader active site and extra disulfide bond in the latter (Kawai et al., 2020). Also, the active form of cutinases is in the form of a Ca^2+^-bound state. There is no Ca^2+^-binding site in the case of PETase. Moreover, serine residue in the catalytic triad of actinomycetes cutinases is replaced with alanine in PETase (Kawai et al., 2020). However, compared to actinomycetes cutinases, PETase is heat liable and act only on lcPET. Considering this, presently researchers are trying to increase the thermostability of PETase and its catalytic efficiency using various protein engineering techniques (Kawai et al., 2020).

The textile or clothing industry is also one of the major producers of PET waste, as it uses polyester as a major raw material. However, the heterogeneous nature of textile waste creates a major hurdle in recycling, as it comprised different types of natural or synthetic plastic wastes. Chemical and mechanical recycling, though, is practiced, but segregation is the first and utmost important step in the recycling of textile wastes. Biocatalytic recycling of textile waste though has potential, but there are limited reports on this aspect. As enzymes are highly specific and thus may target the suitable substrate (PET) in a heterogeneous kind of waste, in this regard, sequential chemical treatment under neutral condition followed by enzymatic treatment for efficient hydrolysis of polyester composed textile waste is reported by Quartinello et al. (2017). Chemical treatment under neutral condition resulted in the production of 85% TA and small oligomers (Quartinello et al., 2017). The oligomers were further hydrolyzed using enzymatic treatment utilizing HiC, yielding 97% of pure TA, available for further recycling. The mixture of PET hydrolases (as mentioned above) could also be used for the biocatalytic conversion of textile polymers into monomers for further recycling. Moreover, compared to other cutinases, actinomycetes cutinases are known to have broad substrate specificity and thus could be used for hydrolysis of a range of polyesters fibers (Kawai et al., 2019).

## Important Factors Affecting Enzymatic PET Hydrolysis

Factors such as crystallinity of PET, temperature, pH of the hydrolysis reactions, buffer strength, and nature of substituent/additives present in plastics (as plasticizers) are some of the factors affecting the enzymatic degradation of PET. These factors can affect the enzymatic PET hydrolysis either by altering the enzyme activity or by inhibiting the accessibility to the ester linkage of the PET.

### Crystallinity(CrI) of PET

Low-CrI PET (lcPET) is easily degradable compared to those with high CrI. High-CrI PET shows high tensile strength and more compactness, and thus, it is difficult for the enzyme to access the ester linkage. Hence, the degradation is hampered. The different enzyme also shows specificity toward particular CrI, for instance, *Pseudomonas mendocina* cutinase (PmC) showed high affinity toward lcPET of 7% CrI compared to *Fusarium solani* cutinase (FsC) (Ronkvist et al., 2009). Cutinase isolated from *T. cellulosilytica* hydrolyzes high-CrI PET at a slower rate compared with lcPET (Herrero Acero et al., 2011). Aliphatic polyesters have low CrI and low *T*_g_ in comparison to semiaromatic polyesters like PET; hence, the latter has limited access to ester linkage (Austin et al., 2018).

### Temperature

Usually high temperature results in better degradation of PET, as it creates flexibility and easy accessibility to ester bonds. Moreover, if the enzyme remains stable up to the *T*_g_ of PET (70–80°C), and then hydrolysis will further be increased. Thus, there is a need for thermostable enzyme, which can optimally work at high temperature or *T*_g_ of PET (70°C). Ronkvist et al. (2009) used *Humicola insolens* cutinase (HiC) to degrade lcPET and observed complete degradation within 94 h of treatment at 70°C, whereas PmC and FsC could only degrade 5% of PET at 50 and 40°C, respectively. Both the enzymes were found to be ineffective with respect to PET degradation at 70°C. Müller et al. (2005) observed only 50% loss in weight of PET at 55°C within 3 weeks of enzymatic treatment using a high concentration of cutinase from *Thermobifida fusca* (*T*f*C*). On the other hand, 97% PET degradation at 80°C within 96 h was detected using HiC with lesser enzyme content (0.13 mg mL^–1^) (Ronkvist et al., 2009), indicating superiority of HiC over TfC considering PET hydrolysis. Indeed, HiC can degrade close to *T*_g_ of PET, indicating a better option for PET degradation.

### pH

Cutinase from most of the organisms are highly active at alkaline pH. However, during continuous enzymatic PET hydrolysis, the media become acidic due to the formation of monomer TPA. This acidic condition affects the enzymatic hydrolysis rate (Ronkvist et al., 2009). Thus, for effective PET hydrolysis, besides thermostability, a broad range of pH stability (toward both alkaline and acidic range) of the enzyme is also one of the prerequisites. Unlike other cutinases, the enzyme from *Thielavia terrestris* was observed to be active at acidic pH in addition to high temperature tolerance, and thus, it is used by Yang et al. (2013) for effective PET degradation.

As TPA-induced acidification of the reaction media is known to inhibit enzymatic activity, increase in buffer strength/concentration lowers this enzyme inhibition (Gamerith et al., 2017a; Furukawa et al., 2019). According to Schmidt et al. (2016), ionic strength and choice of buffer (Tris–HCl, phosphate, MOPS, HEPES) thus affect the enzymatic hydrolysis of PET. Thus, along with pH, the ionic strength of the buffer also affects the PET hydrolysis rate.

### Specificity of the Enzyme

Mostly PET is characterized by an amorphous fraction, which is easily accessed by the enzyme and catalytically converted into oligomers or monomers, whereas the crystalline fraction is more rigid and has limited access by the enzyme. Thus, hcPET has lower degradation rates with hydrolases. Generally, PET hydrolases catalyze endo-type hydrolysis activity by cleaving internal ester bonds of PET, resulting in the formation of oligomers (Kawai et al., 2019). However, Wei et al. (2019) using nuclear magnetic resonance spectroscopy detected both endo- and exo-type hydrolysis activity toward the amorphous region of PET (food-packing container) using recombinant PET hydrolase (thermophilic TfCut2 from *T. fusca*) expressed in *B. subtilis*. With the combination of endo and exo activity, an amorphous region is rapidly hydrolyzed by the recombinant enzyme, resulting in >50% weight loss after 96 h of enzymatic treatment at 70°C. The remaining crystalline structure is hydrolyzed slowly with only endo-chain scission activity, resulting in no detectable weight loss (Wei et al., 2019).

## Methods to Enhance Thermostability of PET Hydrolases

As mentioned previously, enzymatic treatment at high temperature favors the hydrolysis of PET. High temperature above PET *T*_g_ (70°C) increases PET/organic polymers mobility, thereby allowing more accessibility to ester links between monomeric units (Shirke et al., 2018). However, high temperature (>70°C) results in kinetic instability and loss of activity. Various methods, *viz*. use of ionic liquids (Jia et al., 2013) phthalic anhydride and glucosamine hydrochloride (Liu et al., 2002) or through other suitable modifiers like Ca^2+^ (DeSantis and Jones, 1999; Miyakawa et al., 2015) and immobilization on suitable matrices (Kumar et al., 2006; Singh et al., 2013), have been used to increase the thermostability of PET hydrolases. The next section discusses some of these approaches.

### Screening and Sourcing of Heat-Stable Enzymes From Hyperthermophilic Microbes

Thermozymes from thermophilic organisms are one of the important sources of heat-stable enzymes. These enzymes possess various adaptive strategies that help them to function in high temperature. These adaptive strategies are very well covered in the review of Han et al. (2019) and Kumar et al. (2019). These types of natural thermophilic PET hydrolases thus could efficiently be used for the management of PET wastes.

### Immobilization

To increase the catalytic efficiency, stability, specificity, and selectivity of enzymes, immobilization of the enzyme system in suitable matrices is usually carried out (Samak et al., 2020). This enhancement in enzymatic properties is due to favorable structural changes in enzyme as a result of immobilization (Samak et al., 2020). PET hydrolases/cutinases have been immobilized on different matrixes for increasing thermostability. However, these immobilized systems were majorly used for synthesis reactions instead of PET hydrolysis. To date, there are limited reports on PET hydrolysis using immobilized PET hydrolases.

Cutinases from HiC, immobilized on Lewatit VP OC 1600, not only enhance the thermostability at 90°C but also confer the enzyme conformational rigidity in the organic surrounding having low water content (Su et al., 2018). This immobilized HiC was later used for the synthesis of butyl laureate using butanol and lauric acid in organic solvent–rich media. Generally, solubility/rigidity of PET polymers increases/lowers in organic solvents, thereby allowing easy accessibility of enzyme to ester bonds of PET for efficient hydrolysis. Hence, this immobilized HiC could also be used for the hydrolysis of PET. Similarly, Nikolaivits et al. (2017) used cross-linked enzyme aggregates (CLEAs) for the immobilization of cutinases from Fusarium oxysporum. The immobilized cutinases showed enhanced thermostability and the system was efficiently used for the synthesis of short-chain butyrate ester as a flavoring compound. On the other hand, Barth et al. (2016) applied cutinase from T. fusca KW3 (TfCut2) immobilized on SulfoLink coupling resin and free LC-cutinase for enhanced and complete hydrolysis of amorphous PET film at 60°C. This dual enzymatic system resulted in a 2.4 fold high degradation rate at 60°compared to free TfCut2 at 24 h of incubation. Table 3 enlists various immobilization matrixes used for the immobilization of cutinases for increasing thermostability and their applications in synthesis as well as hydrolysis reactions.

**TABLE 3:** Immobilization of cutinases on various matrixes for increasing thermostability and their applications in synthesis and hydrolytic reactions.

Microorganism and enzyme	Matrix interaction	Increase in thermal stability	Hydrolysis/synthesis reactions	References
*Fusarium solani pisi* recombinant cutinase	Silica–covalent bonding	Optimum temperature from 40 to 50∘C	Tricaprylin hydrolysis	Gonçalves et al., 1996
*Fusarium oxysporum*cutinase	Nanoporous gold–polyethyleneimine combination of covalent, electrostatic, and physical adsorption	Maximum activity at 40∘C compared to free enzyme	As adsorbent for removal of contaminants	Zhang et al., 2014
*Thermobifida fusca*KW3 cutinase (TfCut2)	SulfoLink coupling resin–covalent Interaction	Increased stability at 60∘C	Amorphous PET film hydrolysis in combination with LC cutinase	Barth et al., 2016
*Humicola insolens* cutinase (HiC)	EC-EP Sepabeads–covalent interaction	Polycondensation at 70∘C	Synthesis of polyesters from diacids/diesters and linear diols	Ferrario et al., 2016
*Thermobifida cellulosilytica* (Thc_Cut1)	EC-EP Sepabeads– covalent interaction	Polycondensation at 70∘C	Synthesis of polyesters from diacids/diesters and linear diols	Ferrario et al., 2016
*Aspergillus* sp. RL2Ct, cutinase	Biopolymer graft copolymerization– adsorption	Increase by ∼20% at 35∘C	*p-*nitrophenylbutyrate hydrolysis	Kumari et al., 2017
*Fusarium oxysporum* cutinase	Cross-linked enzyme aggregates (CLEA)–non-covalent interaction	Thermostability increased by 10% (50∘C)	Synthesis of short-chain butyrate esters	Nikolaivits et al., 2017
*Thermobifida Cellulosilytica* (Thc_Cut1)	Opal, coral, amber beads chelated with Fe ions via His-tag binding–covalent interaction	57%–78% monomer conversion at 21∘C	Synthesis of aliphatic polyesters	Pellis et al., 2017
*Aspergillus oryzae* cutinase (AoC)	Lewatit VP OC 1600–hydrophobic interaction	AoC activity increased 43% from 40∘C to 70∘C and decreased ∼30% at 80∘C and 90∘C	Butyl laurate synthesis in organic solvent (nonane)	Su et al., 2018
*Humicola insolens* cutinase (HiC)	Lewatit VP OC 1600–hydrophobic interaction	HiC activity increased from 40∘C to 75∘C and decreased ∼15% at 80∘C and 90∘C	Butyl laurate synthesis in organic solvent (nonane)	Su et al., 2018
*Thielavia terrestris* cutinase (TtC)	Lewatit VP OC 1600–hydrophobic interaction	TtC activity increased 60% from 40∘C to 60∘C and showed no significant change at 70∘C, 80∘C, and 90∘C	Butyl laurate synthesis in organic solvent (nonane).	Su et al., 2018

*^∗^Application in removal of contaminants, not hydrolysis and synthesis reactions.*

### Glycosylation of Enzyme

Attachment of polysaccharide chains “glycans” to proteins is termed as glycosylation. Modification of protease through glycosylation resulted in a noticeable effect on kinetics, structure, folding, and stability of enzyme (Goettig, 2016). Glycosylation-based modification has been performed on cellulose (Greene et al., 2015), lipase (Pinholt et al., 2010), and α-amylase (Hu et al., 2019) to achieve enhance enzymatic thermostability. In *Thielavia terrestris*, cutinase glycosylation was introduced that inhibited thermal aggregation (Shirke et al., 2016b). Similarly, glycosylation site engineering was introduced in the cutinase of *A. oryzae* showing the same effect (Shirke et al., 2016a). Leaf and branch compost originated cutinase (LCC) was glycosylated (LCC-G) and compared with non-glycosylated (LCC-NG) (Shirke et al., 2018). Glycosylation helps the protein to reach *T*_g_ temperature of PET without adversely affecting the protein. Aggregation of LCC-NG was observed at 70°C due to hydrophobic interaction, whereas LCC-G showed 10°C higher temperature to achieve the same level of aggregation.

### Use of Surfactants and Additives

The activity of enzymes can be influenced by the application of surfactants. Through binding with the enzymes, surfactants can alter the secondary and tertiary structures or flexibility of enzyme, thereby affecting the enzyme kinetic properties (Rubingh, 1996). In addition to that, the use of surfactants can also improve the dispersibility of PET particles and thus may increase the accessibility of the substrate to enzymes. Chen et al. (2010) in their study recorded increased degradation of PET film (73.65%) using cutinase from *F. solani pisi* in presence of surfactant sodium taurodeoxycholate in the reaction mixture. On the same note, the addition of surfactant alkyl trimethyl ammonium chloride in the reaction media resulted in complete degradation of PET at varied temperature using *Thermobifida fusca* double-mutant cutinase (TfCut2) (Furukawa et al., 2019). Similarly, the use of additives, *viz*. Ca^2+^, Mg^2+^, and hydrophobins (cysteine-rich proteins), has also been used to increase the enzymatic PET hydrolysis (Espino-Rammer et al., 2013; Then et al., 2015; Fukuoka et al., 2016; Joo et al., 2018).

Hydrophobins are cysteine-rich fungal proteins laden with high surface-active substances; it helps in creating interfaces between hydrophobic and hydrophilic phases through their natural tendency of absorption to hydrophobic surfaces (Ribitsch et al., 2015). In this way, they assist in establishing proper interaction of hydrophobic substrates with the active site of enzymes. Several researchers fused hydrophobins with PET hydrolases for increasing PET-hydrolyzing activity (Ribitsch et al., 2015; Puspitasari et al., 2020). As far as Ca^2+^ is concerned, cutinases possess three Ca^2+^-binding sites, and this binding results in activation of the enzyme. Furthermore, Ca^2+^-bound enzyme is prone to less fluctuation compared to the unbound form of an enzyme (Kawai et al., 2020). This stability due to the binding of Ca^2+^ results in higher temperature tolerance and thus helps in PET hydrolysis (Kawai et al., 2020). Furthermore, the fusion of PET hydrolases with the substrate-binding module (SBM) also has been reported to increases PET hydrolysis. Enzymes acting on an insoluble substrate contain SBM for proper interaction with substrates. Altering the surface properties of cutinases through fusion with SBM from other enzymes facilitates the absorption of PET and *vis-à-vis* PET hydrolysis (Kawai et al., 2020). In this regard, Ribitsch et al. (2013) fused cutinase from *Thermomyces cellullosylitica* (Thc_Cut1) separately with the carbohydrate-binding module of cellobiohydrolase I from *Trichoderma reesei* and polyhydroxyalkanoate (PHA)–binding module of polyhydroxyalkanoate depolymerase from *Alcaligenes faecalis*. The catalytic domain of cutinase was linked with SBM through the spacer and thereafter cloned and expressed in *Escherichia coli*. Both fused enzymes showed enhanced hydrophobic interactions with PET and catalytic degradation compared to native cutinase.

### Use of Ultrasonic Waves

Ultrasonic waves are high-frequency waves exceeding 20 kHz and are used to increase the enzymatic PET hydrolysis. Pellis et al. (2016) visualized the effect of ultrasonic waves on enzymatic reaction and observed enhanced PET hydrolysis of various CrI. Sonication of amorphous PET (8% CrI) with Thc_Cut1 (cutinase) during hydrolysis reaction showed an increase of 5.2-fold in TA concentration and 6.6-fold in MHET concentration compared to control. The same reaction with PET of 28% CrI resulted in 2.9-fold increase in TA level. For amorphous PET, better enzymatic hydrolysis was observed with 10 min of sonication, whereas for high-CrI PET, it was observed to be 30 min. According to Pellis et al. (2016), sonication causes changes in the secondary structure of an enzyme, and this unfolding/changes with exposed hydrophobic groups lead to increased enzyme–substrate affinity and *vis-à-vis* enhanced hydrolysis rate. Also, the lesser hydrolytic rate observed in the case of film substrate (PET) compared to amorphous PET is due to less accessibility to the substrate surface in a film.

### Use of Mixture of Enzymes

Use of consortia/mixture of enzymes *viz.* lipases, esterases, and cutinases has been reported by various authors for increasing PET hydrolysis. Carniel et al. (2017) investigated the synergistic study with HiC and CALB. HiC acts as PETase and converts PET to MHET, whereas CALB acts as MHETase converting MHET to TPA or BHET to TPA. The mixture of enzymes causes a 7.7-fold increase in TPA as compared to HiC alone. With synergistic action of enzymes, degradation is found to be effective with hcPET bottles also.

### Engineered Enzymes

In the recent past few years, the most thrilling development is the application of genetic engineering to enzyme technology. A number of enzymatic properties can be altered or improved by genetic engineering to achieve better kinetics of the enzyme and easy downstream processing. This process/technique includes changing amino acid sequences through recombinant DNA mutation. In this regard, double mutation of PETase caused increased PET degradation as a result of more interaction and high binding affinity with the PET (Ma et al., 2018). Similarly, Furukawa et al. (2019) worked with *T. fusca* double-mutant cutinase and observed complete PET degradation.

Site-directed mutagenesis was performed on *T. fusca* cutinase (Tfu_0883), thereby enhancing its activity on PET through substituting bulky amino acid on active site by smaller residue such as alanine (Silva et al., 2011). This provides a less restrained active site to Tfu_0883 and allows better accommodation of PET. Cutinase (Cut190^∗^) from *S. viridis* AHK190 is the only cutinase exhibiting inactive or active state based on binding of Ca^2+^ in its binding site, where it (Ca^2+^) plays activation and thermal stabilization functions (Oda et al., 2018). Cut190^∗^ has three Ca^2+^-binding sites having different amino acids at each site. A mutant at these sites has shown the highest *T*_m_ values and highest PET degradation of (0.25-mm-thick film of amorphous PET, CrI of 6.3%) more than 30% at 70°C (Oda et al., 2018). Recently, Tournier et al. (2020) modified earlier known as leaf-branch compost cutinase (LCC) using site-specific saturation mutagenesis through altering 11 amino acid residues in the enzyme active site. The resulting modified enzyme showed more than 90% depolymerization of PET into monomers within 10 h of treatment. According to the authors, this is the fastest PET-hydrolyzing enzyme reported so far.

## Constraints With Enzymatic PET Hydrolysis

Although PET hydrolases, *viz.* cutinase, PETase, lipase, and esterase, have been reported to hydrolyze PET in various forms, but the following constraints usually lower the efficacy of the process:

(1) Low catalytic turnover due to limited accessibility to active sites as a result of high CrI of the substrate.
(2) Inhibition by MHET or intermediate metabolites.
(3) Kinetic instability and loss of activity at the temperature above PETs *T*_g_ (70°C) (Shirke et al., 2018).
(4) During biodegradation/hydrolysis, solution gets acidic due to end products leading to inactivation of enzyme and hence slower reaction rate.

## Conclusion and Prospects

Among various available methods known for recycling PET, enzymatic methods are considered an environmentally safer and efficient method for managing the PET wastes. Enzyme prominently cutinases are proved to be quite effective in PET hydrolysis. Enzymatic hydrolysis of PET is favored at high temperature, but limited PET hydrolases are known to be active at high temperature. Thus, kinetic instability of enzymes at high temperature is one of the major constraints toward PET hydrolysis. In addition to that, high CrI and low solubility of the substrate and acidic pH of the media during hydrolysis are some of the major factors limiting the hydrolysis rate. However, various modifications such as glycosylation and immobilization of enzymes could be used to enhance thermostability and enzyme activity toward PET. High-CrI PET has higher *T*_g_, and for hydrolysis of these PET, there is a need for enzymes that are stable at such high temperature. Most industries use high CrI plastics, which have high tensile strength and stiffness and are less affected by solvents. Recycling such plastics has become a tedious problem. Additionally, enzymatic treatment of mixed wastes arising from the textile industry too is a problem, as this enzyme needs to have broad substrate specificity. If the search for a novel thermostable PET-hydrolyzable enzyme capable of hydrolyzing hcPET with broad substrate specificity is fulfilled, then this will ultimately help in the overall curbing of plastic pollution in a sustainable pattern.

## Author Contributions

AM and AB designed and wrote the manuscript, while SK conceived the idea and critically review the manuscript with valuable inputs. All authors contributed to the article and approved the submitted version.

## Conflict of Interest

The authors declare that the research was conducted in the absence of any commercial or financial relationships that could be construed as a potential conflict of interest.
